# Climate change, air pollution and chronic respiratory diseases: understanding risk factors and the need for adaptive strategies

**DOI:** 10.1265/ehpm.24-00243

**Published:** 2025-01-30

**Authors:** Jiayu Xu, Zekang Su, Chenchen Liu, Yuxuan Nie, Liangliang Cui

**Affiliations:** 1 Vanke School of Public Health, Tsinghua University, Beijing, 100084, China; 2 School of Public Health, Chengdu Medical College, Chengdu, 610500, China; 3 Jinan Mental Health Center, Jinan, 250309, China; 4 School of Public Health, Bengbu Medical University, Bengbu, 233030, China

**Keywords:** Climate change, Air pollution, Extreme weather events, Respiratory health, Vulnerability

## Abstract

Under the background of climate change, the escalating air pollution and extreme weather events have been identified as risk factors for chronic respiratory diseases (CRD), causing serious public health burden worldwide. This review aims to summarize the effects of changed atmospheric environment caused by climate change on CRD. Results indicated an increased risk of CRD (mainly COPD, asthma) associated with environmental factors, such as air pollutants, adverse meteorological conditions, extreme temperatures, sandstorms, wildfire, and atmospheric allergens. Furthermore, this association can be modified by factors such as socioeconomic status, adaptability, individual behavior, medical services. Potential pathophysiological mechanisms linking climate change and increased risk of CRD involved pulmonary inflammation, immune disorders, oxidative stress. Notably, the elderly, children, impoverished groups and people in regions with limited adaptability are more sensitive to respiratory health risks caused by climate change. This review provides a reference for understanding risk factors of CRD in the context of climate change, and calls for the necessity of adaptive strategies. Further interdisciplinary research and global collaboration are needed in the future to enhance adaptability and address climate health inequality.

## 1. Introduction

Climate change refers to long-term shifts or alterations in weather conditions and patterns of extreme weather events (EWEs), such as temperature, precipitation, wind patterns, and other aspects of the earth’s climate system [1]. Adverse outcome caused by climate change includes intensified air pollution, rising temperatures, and increased frequency of EWEs [2]. Overall, climate change arises from two main factors: anthropogenic activities, such as greenhouse gas (GHGs) emissions, fossil fuel combustion, agriculture, deforestation and natural processes, including solar radiation fluctuations and volcanic eruptions [3, 4]. Global warming, a key component of climate change, is marked by the rise in earth’s average surface temperature, primarily driven by increased atmospheric concentrations of carbon dioxide (CO_2_) and other GHGs, which enhance the greenhouse effect and trap heat [5]. Besides, continued emission of GHGs will cause further warming and long-lasting changes in the climate system, such as precipitation patterns, more frequent and intense EWEs, and shifts in seasonal temperatures [6]. Notably, climate change can exert detrimental impacts on public health and wellbeing, specifically causing significant damage to the respiratory system [7, 8].

Climate change has significantly intensified air pollution concerns. Air pollution consists of particulate matter (PM) and gases such as nitrogen oxides (NOx), sulfur dioxide (SO_2_), and ozone (O_3_) in the air [9]. When the amount of these pollutants released into the atmosphere exceeds its self-purification capacity, leading to an increase in pollutant concentrations, it results in direct, indirect, or potential adverse effects on human health and the ecological environment [9]. Elevated temperatures contribute to a surge in heatwaves and degrade air quality further. For example, higher temperatures and more intense sunlight can accelerate the production of ground-level O_3_ [10]. Moreover, climate change is contributing to more frequent and severe wildfires, which produce harmful smoke and particulates [11]. It is estimated that under a 2 °C warming scenario, wildfire frequency is projected to increase significantly in many regions [12]. By altering temperature, precipitation, and wind patterns, as well as increasing the likelihood of EWEs, climate change creates conditions that are more conducive to wildfire occurrence, spread, and intensity [13, 14]. Specifically, higher temperatures and prolonged droughts dry out vegetation, making it more flammable, while changing precipitation patterns can affect the availability and condition of burnable material. Furthermore, shifting wind patterns can facilitate the rapid spread of fires [15, 16].

The World Health Organization identifies chronic respiratory diseases (CRD) as one of the four principal chronic conditions, responsible for an estimated 7.5 million fatalities annually, which is about 14% of global deaths each year [17], causing tremendous economic and human burden. Data from the 2017 Global Burden of Diseases, Injuries, and Risk Factors Study (GBD) indicates that chronic respiratory conditions continue to be a predominant source of mortality and impairment globally [18]. Chronic obstructive pulmonary disease (COPD) and asthma are among the most frequently diagnosed chronic respiratory diseases [19]. The occurrence and progression of CRD are influenced by multiple factors, among which environmental factors play a significant role [20].

Although previous studies have summarized the association and mechanism of action between air pollution and CRD, there is still a lack of systematic review about the impact of climate change on respiratory health from the perspective of combined exposure to the atmospheric environment, which refers to the physical and chemical characteristics of the atmosphere on which organisms depend, including air pollutants, adverse meteorological conditions and atmospheric allergens. This review summarizes the respiratory health effects associated with climate change, including potential pathophysiological mechanisms, influencing factors, and health inequalities, etc. The purpose of this study aims to provide reference for understanding the risk factors of chronic respiratory diseases in the context of climate change, and calls for the necessity of adaptive strategies.

## 2. Materials and methods

The methodology for this literature review is structured to systematically identify recent studies and conduct a comprehensive evaluation. The CRD included in this study primarily consist of COPD (ICD-10, J44) and asthma (ICD-10, J45–J46). Searches were conducted between January, 1 and April, 30 2024 using PubMed and Web of Science, with a combination of the following search terms: “climate change”, “air pollution”, “global warming”, “extreme weather events”, “sandstorms”, “thunderstorm”, “heatwave”, “respiratory health”, “respiratory diseases”, “asthma”, “chronic obstructive pulmonary disease”, “particulate matters (PM)”, “ozone (O_3_)”, “nitrogen dioxide (NO_2_)”, “sulfur dioxide (SO_2_)”, as well as disease-specific keywords for the conditions discussed in this review. Publications from 2000 to 2024 were considered for inclusion, with particular emphasis on those published within the last five years. Articles were excluded if they were not peer-reviewed, not available in English, or did not address the effects of climate change on at least one of the chronic respiratory diseases covered in this review. Meeting abstracts, letters, news articles, and comments were not included in the review.

## 3. The influence of climate change on the atmospheric environment

Air pollution and climate change are intricately connected, with their origins, impacts, and mitigation strategies deeply interwoven [21]. From a causative standpoint, air pollution and climate change are generally driven by similar sources, particularly the combustion of fossil fuels [22]. This process primarily releases CO_2_, methane (CH_4_), and nitrous oxide (N_2_O), which are potent greenhouse gases contributing to global warming [23]. Additionally, it emits SO_2_, NOx, PM, and volatile organic compounds (VOCs), which contribute to air pollution and can form secondary pollutants such as ground-level O_3_ and smog [24]. Notably, certain air pollutants, particularly black carbon, serves as both an air pollutant and a potent short-term climate forcer by absorbing solar radiation, thereby accelerating local and global warming [25, 26]. Climate change can also exacerbate air pollution by intensifying EWEs like droughts, heatwaves, and wildfires, which contribute to higher pollutant concentrations [12]. Therefore, air pollution control policies and climate change mitigation strategies are often synergistic, offering dual benefits when implemented together.

Table 1 summarized the air pollution issues and countermeasures in major countries during different historical periods. It is worth noting that different countries or regions have adopted targeted control strategies for major air pollutants. For example, the photochemical smog issue in Los Angeles was primarily addressed through the reduction of vehicle emissions and industrial emission controls, targeting the reduction of NOx, VOCs and O_3_ [27]. In contrast, the haze events in China were primarily mitigated by the transition to cleaner energy sources (with a focus on reducing coal usage) and the implementation of stricter vehicle emissions standards alongside the promotion of electric vehicles in order to address particulate matter pollution [28].

**Table 1:** Summary of air pollution issues and countermeasures in major countries during different historical periods

**Region/Country**	**Period**	**Major pollutants**	**Related health issues**	**Strategies implemented**
**Los Angeles, ** **U.S.**	1940s to 1960s	O_3_; NOx; VOCs; Particulate matter	Respiratory diseases (e.g., asthma, bronchitis); Eye irritation; Sore throat, coughing; Reduced cardiopulmonary function	Implementation of the Clean Air Act (1970) to strengthen air quality standards; Reduction of vehicle emissions (e.g., catalytic converters); Promotion of public transportation and industrial emission controls; Air quality monitoring, and smog control and warning mechanisms
**London, UK**	1950s	SO_2_; Particulate matter; Coal smoke	Respiratory problems (e.g., bronchitis, pneumonia); Heart failure; Eye irritation; Premature deaths	Implementation of the Clean Air Act (1956) to restrict coal burning in urban areas; Promotion of cleaner energy sources; Improved air quality monitoring; Control industrial emissions
**Japan**	1960s to 1970s	SO_2_; NOx; Particulate matter	Yokkaichi Asthma; Respiratory diseases caused by SO_2_ and PM, including bronchitis and asthma; Increased rates of respiratory infections and heart disease	Enactment of laws like the Basic Law for Environmental Pollution Control (1967) and Air Pollution Control Act (1968); Establishment of Environment Agency in 1971 to oversee pollution control; Stricter regulations on industrial emissions and waste management
**Belgium**	1930s	SOx; Particulate matter; NOx	Respiratory problems, such as severe bronchitis and asthma attacks; Cardiovascular complications, including heart failure due to dyspnea; Eye and throat irritation	Increased monitoring of air pollution in industrial areas; Awareness of public health risks from industrial emissions; Enhanced enforcement of air pollution prevention laws.
**China**	Early 2000s, with severe episodes in 2013 and 2015	Particulate matter (PM_2.5_ and PM_10_); SO_2_; NOx; O_3_	Increased cases of respiratory diseases, such as asthma and chronic bronchitis; Higher incidence of cardiovascular diseases; Increased hospital admissions, especially among vulnerable populations (elderly, children, those with pre-existing conditions)	Implementation of Air Pollution Prevention and Control Action Plan (2013) to reduce PM levels by tightening emissions standards; Transition to cleaner energy sources (reducing coal usage); Stricter vehicle emissions standards and the promotion of electric vehicles; Strengthened monitoring and enforcement of pollution controls; Public awareness campaigns and encouragement of individual actions to reduce pollution (e.g., limiting vehicle use).

Abbreviations: PM_2.5_, particulate matter ≤2.5 µm in aerodynamic diameter; PM_10_, particulate matter ≤10 µm in aerodynamic diameter; NOx, nitrogen oxides; VOCs, volatile organic compounds; O_3_, ozone; SO_2_, sulfur dioxide; SOx, sulfur oxides; CO, carbon monoxide.

Table 2 summarized the major pollutants and key contributing factors in countries or regions where air pollution remains severe. It has been reported that air pollution significantly impacts human health in Africa, one of the world’s most affected regions. In 2019, a study utilizing WHO Global Health Observatory and GBD data estimated that Africa, severely affected by air pollution, accounted for 1.1 million premature deaths or 16.3% of the worldwide total [29]. Outdoor air pollution in Africa arises from various sources including dust storms, waste incineration, biomass burning, industrial emissions, etc. Notably, dust storms significantly exacerbate air pollution in Africa, impacting air quality and human health [30]. In addition, other countries such as India, Pakistan, and Bangladesh are also confronting significant challenges related to air pollution within the context of climate change, with their efforts to implement efficacious strategies to mitigate these issues proving deficient [31–33].

**Table 2:** Summary of major pollutants and key contributing factors in countries or regions with persistent air pollution

**Region/Country**	**Main pollutants**	**Key factors contributing to air pollution**
**Africa**	Particulate matter (PM_2.5_ and PM_10_); SO_2_; NOx; CO; VOCs	Dust storms; Biomass burning; Waste incineration; Agricultural burning; Industrial emissions
**India**	Particulate matter (PM_2.5_ and PM_10_); SO_2_; NOx	Industrial emissions; Vehicular exhaust; Biomass burning; Fossil fuel combustion
**Pakistan**	Particulate matter (PM_2.5_ and PM_10_); NOx; SO_2_; VOCs; O_3_	Industrial emissions; Agricultural burning; Construction dust; Fossil fuel combustion
**Bangladesh**	Particulate matter (PM_2.5_ and PM_10_); SO_2_; NOx; CO; VOCs	Vehicular exhaust; Burning of biomass and agricultural waste; Industrial emissions; Construction activities; Wood and coal burning

Abbreviations: PM_2.5_, particulate matter ≤2.5 µm in aerodynamic diameter; PM_10_, particulate matter ≤10 µm in aerodynamic diameter; NOx, nitrogen oxides; VOCs, volatile organic compounds; SO_2_, sulfur dioxide; CO, carbon monoxide.

### 3.1 Climate change is closely linked to the exacerbation of ozone pollution

Climate change can affect the transport and chemical reactions of air pollutants. For example, rising temperatures can lead to an increase in ground-level O_3_ concentrations by accelerating the chemical reactions between VOCs and NOx in the presence of sunlight [34, 35]. Especially in the troposphere O_3_ is considered a pollutant, which means global warming may exacerbate the O_3_ pollution in the ground layer [36]. On the other hand, climate change can indirectly affect O_3_ levels through its impact on atmospheric circulation patterns [37]. Changes in wind patterns and temperature gradients can alter the transport of O_3_ between different layers of the atmosphere, potentially impacting stratospheric O_3_ concentrations [38]. Furthermore, climate change can also impact stratospheric O_3_ through water vapour cycle. As climate change progresses, the cooling of the polar stratosphere may slow the recovery of the O_3_ layer [39]. Notably, climate change can exacerbate EWEs, which could further affect the stability of the O_3_ layer. A reduction in stratospheric O_3_ results in increased ultraviolet (UV) radiation reaching the earth’s surface, heightening the risk of health concerns and also negatively affecting ecosystems [40, 41].

### 3.2 Climate change elevates the levels of particulate matters and other pollutants

Climate change can exacerbate air pollution by influencing the distribution and concentration of particulate matters and other air pollutants [42]. Particulate matter plays a significant role in the air quality deterioration resulting from a range of both natural and human-induced actions [43]. According to the aerodynamic diameter of particulate matters, it can be categorized as PM_10_ (aerodynamic diameter <10 µm), PM_2.5_ (aerodynamic diameter <2.5 µm), and ultrafine particles (aerodynamic diameter <100 nm) [44]. In the context of climate change, the frequency of EWEs, such as wildfires and dust storms has increased, which can lead to large amounts of PM release into the atmosphere, and then be carried over long distances [45]. Besides, higher temperatures enhance the formation of secondary PM from atmospheric pollutants like NOx and VOCs, while altered weather patterns can reduce pollutant dispersion, further raising PM levels. Climate change can also directly influence local and regional air quality by altering chemical reaction rates, boundary layer heights that impact the vertical mixing of pollutants, and synoptic airflow patterns that control pollutant transport [46]. Therefore, the exacerbation of air pollution caused by climate change is global, which means increased levels of PM can happened in regions that are not directly affected by EWEs. Additionally, climate change can also affect the sources and emissions of other air pollutants, e.g. NO_2_, carbon monoxide (CO), SO_2_ and VOCs [45].

### 3.3 Climate change affects atmospheric allergen levels

Climate change significantly impacts both the release and distribution of allergens. For example, it can alter the timing, duration, and intensity of pollen seasons, leading to prolonged plant growth seasons and thereby promoting pollen release. Besides, elevated atmospheric CO_2_ levels have been shown to enhance plant photosynthesis and reproductive processes, resulting in increased pollen production. Consequently, this exacerbates the exposure to allergens and intensifies allergic reactions [47]. Notably, climate change may also lead to changes in the region of plants, resulting in an expansion or alteration of the distribution range of allergens [48]. In particular, pollen sufferers may face more types of allergens as new plant species enter their regions. Additionally, high humidity and air pollutants induced by climate change can exacerbate effects of atmospheric allergens by irritating the respiratory system, making individuals more susceptible to allergic reactions.

## 4. The impact of climate change on chronic respiratory diseases

As a result of climate change, patterns of air pollution are shifting in many urban regions globally, exerting a considerable impact on respiratory health, both as a standalone factor and in combination with meteorological conditions [49]. Furthermore, EWEs such as sandstorms, wildfires, thunderstorms, along with adverse meteorological conditions, including temperature variability and high humidity environments, also exert adverse impact on respiratory health [50–52].

### 4.1 The impact of air pollution on CRD

Results from a systematic review and meta-analysis which included 436 studies globally indicated that per 10 µg/m^3^ increase in PM_2.5_ levels correlates with a higher incidence of COPD, with a pooled hazard ratio (HR) of 1.18 (95% CI: 1.13, 1.23) [53]. According to the WHO Global Air Quality Guidelines, the maximum 8-h O_3_ concentration standard is 100 µg/m^3^ [54]. Findings from a meta-analysis revealed that an increase of 10 µg/m^3^ in maximum 8-h O_3_ concentration correlated with a 0.84% (95% CI: 0.09%, 1.59%) rise in hospital admissions for COPD [55]. Additionally, several epidemiological studies have indicated that patients with asthma were particularly vulnerable to rises in ground-level O_3_ [56, 57]. Fang X et al. found that male and older asthma patients may be more vulnerable to O_3_ especially in the warm season [57].

PM_2.5_ from wildfire was also positively correlated to adverse respiratory health outcomes. For example, results from Alman, BL et al. showed positive relationships between PM_2.5_ and COPD (OR = 1.05, 95%CI: 1.02, 1.08 per 5 µg/m^3^) and asthma (OR = 1.04, 95% CI: 1.02, 1.06 per 5 µg/m^3^) [58]. Furthermore, particulate matter in outdoor air pollution was identified as Group I carcinogen by the International Agency for Research on Cancer (IARC) [59]. Prolonged exposure to elevated levels of particulate matter can elevate the risk of numerous health problems, with CRD being particularly prominent. Although the wildfire usually maintains relatively short duration, the released smoke and PM will persist in the atmosphere for a long time, thereby affecting a potential large population. In addition, temperature rise can also increase the release of VOCs, which can stimulate respiratory mucosa, cause respiratory diseases and allergic reactions.

### 4.2 The effects of extreme weather events on CRD

Climate change exacerbates the frequency and intensity of drought and extreme high temperatures, while also affecting precipitation patterns and amounts, thereby increasing the risk of forest fires. A systematic review and meta-analysis revealed that a rise in EWEs elevated the risk ratios of asthma occurrences to 1.19 (95% CI: 1.08, 1.32) in children and 1.29 (95% CI: 0.98, 1.69) in female, respectively [60]. Previous evidence indicated that particulate matter levels typically exceeded the standard pollution levels of the area during wildfire incidents [61]. In summer 2010, a prolonged heatwave occurred in the Russian, accompanied by catastrophic wildfires. Compared to the same period, a rise in respiratory disease-related deaths, amounting to 339, was observed in Moscow (RR = 2.05, 95% CI: 1.80–2.39), which demonstrated a combined impact of heat and air pollution on mortality [62]. Meanwhile, changes in overall climate patterns have led to soil drying and water depletion in some areas, increasing the frequency and intensity of sandstorms. Long-term exposure to sandstorm environments may lead to the development and aggravation of COPD, etc. [52, 63].

Climate change is closely related to extreme precipitation events such as heavy rainfall, flood and hurricane [64]. Patients with CRD (such as COPD, asthma, etc.) already suffered from chronic inflammation of the respiratory tract, and are easily affected by moisture and humid environments. Heavy rainfall also increases the growth of indoor fungi, leading to aggravated allergic reactions and respiratory infections in CRD patients. For example, Hayes D Jr et al. found a strong correlation between mold sensitization and asthma, with humidity identified as a worsening factor for patients suffering from allergic asthma [65]. In addition, while humidity is not a direct cause for interstitial pneumonia, it facilitates the growth of mold and microorganisms, which upon inhalation, may exacerbate inflammation or lead to secondary infections. In hypersensitivity pneumonitis, elevated humidity accelerates mold and fungal spore proliferation, with exposure to these allergens frequently inducing symptoms such as coughing and dyspnea [66]. High humidity can also promote the growth of bacteria and viruses, thereby increasing the risk of respiratory tract infections.

It is predicted that atmospheric alterations related to thunderstorms are likely to become more intense in face of climate change [67]. Thunderstorm-induced asthma refers to the onset of sudden asthma attacks that happen immediately following a thunderstorm, often referred to as “thunderstorm asthma” [68]. During thunderstorms, high winds and electrical activity can cause pollen grains to rupture into smaller particles, which can be inhaled and penetrate deeper into the airway. Pollen allergens can trigger the release of pro-inflammatory and immunomodulatory mediators, thereby accelerating the onset of IgE-mediated sensitization and allergic reactions [47]. This process can precipitate early asthmatic reactions, following the inhalation of elevated concentrations of aeroallergens [69]. Additionally, the increased humidity and cooler air associated with the thunderstorm may exacerbate airway sensitivity, leading to bronchoconstriction and asthma symptoms, especially in individuals with preexisting allergies or asthma [70]. Except for asthma, Eric Zou et al. found that emergency department visits for COPD also significantly increased during thunderstorms period. The primary mechanism underlying thunderstorm-related acute respiratory disease except for asthma may be the preceding increases in the concentration of particulate matter and temperature variation [67].

### 4.3 The impact of adverse meteorological conditions on CRD

Sudden changes in temperature and humidity are also linked to increased emergency department (ED) visits for patients suffering from chronic respiratory disease [71]. Previous evidence showed that a 1 °C rise in maximum temperature variability elevates the risk of asthma by 5.0% globally, with this effect being particularly pronounced among individuals residing in high-latitude regions or those aged between 50 and 70 years [50]. Tran HM et al. found that following adjustments for annual PM_2.5_ levels, socio-demographic index level, smoking prevalence, and geographical regions, a 0.26% rise in deviance percentage of temperature corresponded to reductions in the log of age-standardized mortality rates (LASMR) of COPD by 0.016, 0.017, and 0.014 per 100,000 individuals and in the LASMR of asthma by 0.042, 0.046, and 0.040 per 100,000 individuals across both genders, males, and females, respectively [72]. Besides, for CRD patients, when the barometric pressure decreases suddenly, the pressure of the gas in the lungs also decreases, which may lead to dyspnea and worsen symptoms [73]. Excessive humidity may also pose negative impact on the respiratory system. In high humidity environments, moisture in the air can easily trigger the growth of fungi and mold, such as Aspergillus, Cladosporium, Penicillium and Stachybotrys, which may lead to respiratory allergic reactions and infections, thereby exacerbating symptoms of allergic respiratory diseases [74, 75]. For example, Aspergillus can trigger allergic reactions like asthma, allergic bronchopulmonary aspergillosis, sinusitis and invasive aspergillosis [76, 77]. Cladosporium may result in hay fever-like symptoms, asthma, and allergic rhinitis, with rare cases of lung infections in immunocompromised individuals [78, 79]. Penicillium exposure can cause respiratory symptoms such as asthma, nasal congestion, sinus inflammation and penicilliosis, a lung-focused fungal infection [80].

## 5. Potential pathophysiological mechanistic pathways linking aggravated air pollution and CRD under climate change

### 5.1 Pulmonary inflammation

The augmentation in air pollution attributable to climate change could potentially intensify the inflammatory response within the pulmonary system. PM_2.5_ could potentially facilitate the progression of COPD by damaging pulmonary function and aggravating lung injury, with potential underlying mechanisms associated with inflammation [81]. Research from Zhao C et al. found that the soluble extract of PM_2.5_ could trigger an increase in pro-inflammatory factor expression by stimulating the nuclear factor kappa B (NF-κB) and mitogen-activated protein kinase (MAPK) signalling pathways. This was coupled with a decrease in airway barrier functionality, contributing to the aggravated progression of asthma [82]. Wang L et al. also found that PM_2.5_ could aggravate airway inflammation in asthmatic mice by activating NF-κB via myeloid differentiation primary response 88 (MyD88) signaling pathway [83]. Acute O_3_ exposure could also induce airway hyperreactivity and neutrophilic inflammation [84]. Besides, air pollution can directly affect the recruitment of inflammatory cells. For instance, a classic research identified that increased levels of O_3_ can stimulate an amplified inflammatory response, characterized by a rise in the proportion of neutrophils and total protein concentrations in bronchoalveolar lavage in asthmatic patients compared to non-asthmatic ones [85].

### 5.2 Immune disorders

Air pollution leads to not only the recruitment of granulocytes in the airway, but also the influx of immune-modulatory cells exhibiting altered cell surface phenotypes associated with antigen presentation [86]. For instance, exposure to O_3_ not only results in an increased presence of dendritic cells and monocytes exhibiting elevated levels of CD14, CD86, human leukocyte antigen-DR isotype (HLA-DR), but also notably escalates the count of neutrophils and myeloperoxidase levels in airway lavages [85, 87]. Exposure to PM_2.5_ can lead to a decrease in the expression of programmed death-ligand 1 (PD-L1) in the lungs, consequently disrupting the establishment of immune tolerance and ultimately leading to allergic airway inflammation [88]. PM_2.5_ can alco exacerbate effects of asthma by regulating transient receptor potential ankyrin 1 (TRPA1), transient receptor potential vanilloid 1 (TRPV1) and the relevant inflammation mediators, such as interleukin (IL)-13, prostaglandin D2 (PGD2) [89]. Animal study showed that exposure to SO_2_ can alter the inflammatory and immune responses in the airways of asthmatic rats by enhancing the expression of pro-inflammatory cytokines and disrupting the Th1/Th2 balance, potentially contributing to an elevated risk of asthma [90].

### 5.3 Oxidative stress

Air pollutants can induce potent oxidative stress responses, which not only leads to cellular damage through the production of reactive oxygen species (ROS), but also disrupts the body’s antioxidant defense mechanisms. Prolonged exposure to air pollutants can trigger the activation of oxidative pathways, leading to cellular death and persistent inflammation in the airways, culminating in emphysema [91]. For example, PM_2.5_ exposure can activate nuclear factor erythroid 2-related factor 2 (Nrf2) related signaling pathways and facilitate lung injury [92]. Xia R et al. found that PM_2.5_ could promote apoptosis of alveolar epithelial cells via targeting ROS/P38 signaling pathway and thus lead to emphysema in mice [93]. Li N et al. found that PM_2.5_ could contribute to pulmonary epithelial senescence and ferroptosis by regulating USP3-SIRT3-P53 axis [94]. Results from Havet A et al. indicated a direct positive correlation between O_3_ exposure and persistent asthma (OR = 1.68, 95%CI: 0.57, 7.25), further elucidating the role of oxidative stress in the relationship between air pollution and chronic asthma [95].

Overall, considerable previous research attention has been devoted to elucidating the pathophysiological mechanisms linked to the intensification of air pollution with CRD. However, investigations into the potential biological pathways of EWEs and unfavorable meteorological conditions remain relatively limited, which highlights the need for more comprehensive studies to clarify the systematic adverse outcome pathway of respiratory health impacts associated with changing climate.

## 6. Potential factors affecting CRD caused by climate change

### 6.1 Individual behavior and adaptability

**Fig. 1:**
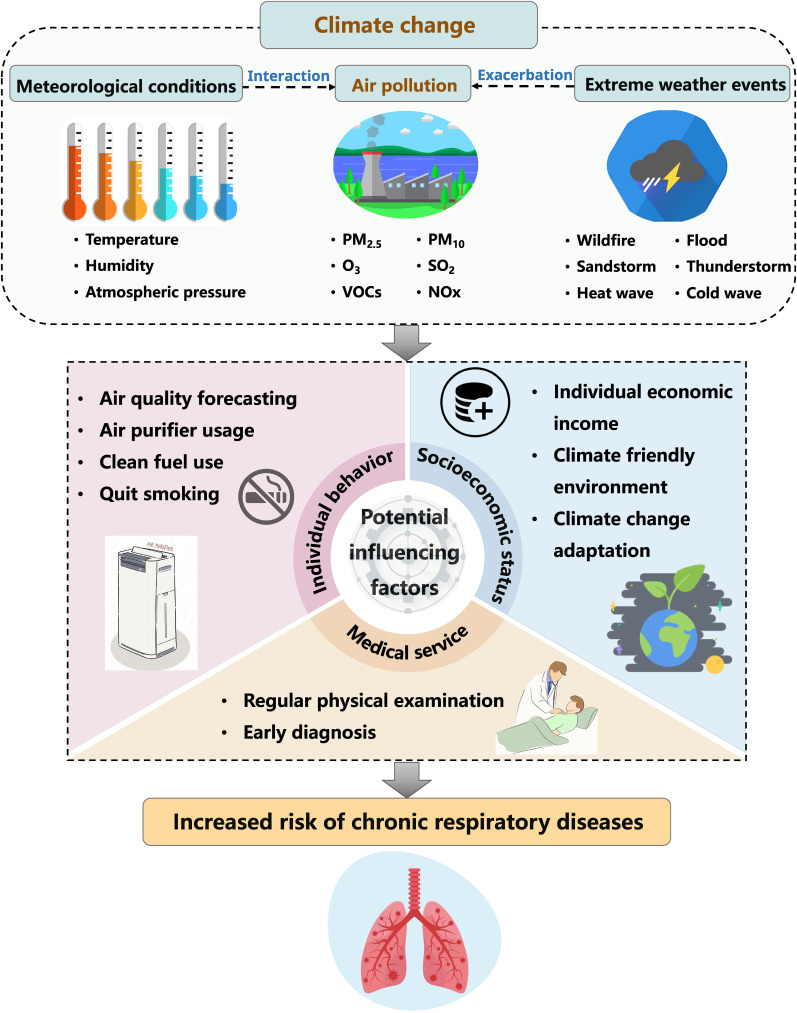
Potential risk and influencing factors linking climate change and chronic respiratory diseases

Individual behavior can pose substantial effects on reducing exposure to air pollution [96]. For example, personal adaptive behavioral such as avoiding cooking with solid fuels, ensuring proper ventilation and separation of cooking areas, and utilizing air purifiers equipped with high-efficiency particulate air filters, can significantly mitigate the respiratory health hazards associated with air pollution under climate change (Fig. 1) [97]. Conversely, smoking related airway inflammation can exacerbate the impact of O_3_ exposure on blood oxygen saturation in COPD patients [98]. It is predicted that outdoor physical activities in areas with high air pollution can expose individuals to harmful pollutants, potentially leading to respiratory health issues. Research conducted by Tikkakoski, AP et al. demonstrated a significant correlation between exercise-induced bronchoconstriction and particulate matter as well as air humidity in preschool-aged children [99]. However, Chen, L et al. indicated that regular physical activity could lower the risk of COPD, and such protective benefits were not influenced by exposure to ambient PM_2.5_ [100]. Therefore, the interaction between physical activity and air pollution on the incidence of CRD still needs further exploration.

### 6.2 Economic development and adaptability to climate change

Limited by the level of economic development in some developing countries or regions, the ability to adapt to climate change is relatively poor in vulnerable areas [101]. Lack of adaptation measures and coping abilities can more easily lead to respiratory health problems [102]. Specifically, factors such as industrialization, traffic congestion, energy consumption and domestic pollution can also exacerbate the impact on respiratory health [103].

### 6.3 Medical services and health examinations

It is difficult for people with limited medical resources and physical examination to detect early symptoms of chronic respiratory diseases, making them more susceptible to the impact of climate change [104]. The fair allocation and comprehensive coverage of health resources are crucial for ensuring health equity and addressing the health impacts of climate change [105]. Therefore, it is necessary to increase investment in medical resources, strengthen health education, and improve climate change adaptation capabilities.

## 7. The inequalities of respiratory health caused by climate change

Climate change has exacerbated health inequality, making vulnerable populations (such as the elderly, children and impoverished individuals) more susceptible to the negative impacts of climate change [106–109]. For example, EWEs such as heatwaves, wildfires and sandstorms can lead to increased health risks for children’s respiratory system [110]. Besides, the impact of climate change on respiratory health is more significant in patients with underlying cardiovascular and pulmonary diseases [111, 112]. People living in regions lacking the ability to respond to EWEs and provide health infrastructure, such as well-equipped sanitation facilities and medical services, are also facing health inequality associated with climate change [113]. Previous studies have shown that the health effects related to heat exposure vary greatly due to geographical regions and socio-economic status [114]. For example, desert regions in Africa face challenges such as fragile ecosystems, lagging economic development, weak sanitation facilities, and limited risk resistance [115]. Climate change has further exacerbated these issues. The increased concentration of particulate matter and other pollutants caused by drought and desertification can directly harm the respiratory system, making the impact on respiratory health particularly more prominent [116].

## 8. Adaptive strategies for respiratory health risks in response to climate change

As climate change progressively intensifies, it is crucial to address these interconnected issues of air pollution and climate change to protect both global environment and public health. Firstly, it is necessary to adopt policies that focus on simultaneous pollution reduction and carbon mitigation, such as promoting renewable energy to replace fossil fuels, which not only reduces carbon dioxide emissions but also decreases air pollutants. The introduction of carbon pricing strategies, including carbon taxes or cap-and-trade mechanisms, could offer financial motivations for companies to decrease carbon emissions and invest in more environmentally friendly technologies. Secondly, it is of vital importance to raise public awareness of the association between climate change and respiratory health through health education and publicity, and further guide them to adopt positive health behaviors and individual protective measures [117]. Finally, in order to address the issues of respiratory health inequality, the international community should strengthen cooperation and take comprehensive measures, such as increasing climate change adaptation capacity, providing health services and infrastructure support, promoting sustainable development and fair and just health rights [118].

## 9. Conclusions

This review provided epidemiological evidence, potential influencing factors and pathophysiological mechanisms underlying the increased risk of chronic respiratory diseases associated with climate change, and calls for the necessity of adaptive strategies. In the future, it is necessary to strengthen interdisciplinary research in the fields of climate change and respiratory health, in order to protect vulnerable populations and improve climate change response capabilities, thereby promoting the climate health equity.

## Declarations

### Availability of data and material

No data was used for the research described in the article.

## References

[1] Wu X, Lu Y, Zhou S, Chen L, Xu B. Impact of climate change on human infectious diseases: Empirical evidence and human adaptation. Environ Int. 2016;86:14–23. 26479830 10.1016/j.envint.2015.09.007

[2] Lothrop N, Lopez-Galvez N, Canales RA, O’Rourke MK, Guerra S, Beamer P. Sampling Low Air Pollution Concentrations at a Neighborhood Scale in a Desert US Metropolis with Volatile Weather Patterns. Int J Environ Res Public Health. 2022;19(6). 10.3390/ijerph19063173 PMC8949442 35328861

[3] Abatzoglou JT, Williams AP. Impact of anthropogenic climate change on wildfire across western US forests. Proc Natl Acad Sci U S A. 2016;113(42):11770–5. 27791053 10.1073/pnas.1607171113 PMC5081637

[4] Aubry TJ, Staunton-Sykes J, Marshall LR, Haywood J, Abraham NL, Schmidt A. Climate change modulates the stratospheric volcanic sulfate aerosol lifecycle and radiative forcing from tropical eruptions. Nat Commun. 2021;12(1):4708. 34385437 10.1038/s41467-021-24943-7 PMC8360950

[5] Filonchyk M, Peterson MP, Zhang L, Hurynovich V, He Y. Greenhouse gases emissions and global climate change: Examining the influence of CO(2), CH(4), and N(2)O. Sci Total Environ. 2024;935:173359. 38768722 10.1016/j.scitotenv.2024.173359

[6] Moore FC, Lacasse K, Mach KJ, Shin YA, Gross LJ, Beckage B. Determinants of emissions pathways in the coupled climate-social system. Nature. 2022;603(7899):103–11. 35173331 10.1038/s41586-022-04423-8

[7] Helldén D, Andersson C, Nilsson M, Ebi KL, Friberg P, Alfvén T. Climate change and child health: a scoping review and an expanded conceptual framework. Lancet Planet Health. 2021;5(3):e164–75. 33713617 10.1016/S2542-5196(20)30274-6

[8] D’Amato G, D’Amato M. Climate change, air pollution, pollen allergy and extreme atmospheric events. Curr Opin Pediatr. 2023;35(3):356–61. 36917187 10.1097/MOP.0000000000001237

[9] Hassoun Y, James C, Bernstein DI. The Effects of Air Pollution on the Development of Atopic Disease. Clin Rev Allergy Immunol. 2019;57(3):403–14. 30806950 10.1007/s12016-019-08730-3 PMC8215519

[10] Ayejoto DA, Agbasi JC, Nwazelibe VE, Egbueri JC, Alao JO. Understanding the connections between climate change, air pollution, and human health in Africa: Insights from a literature review. J Environ Sci Health C Toxicol Carcinog. 2023;41(3–4):77–120. 37880976 10.1080/26896583.2023.2267332

[11] Orru H, Ebi KL, Forsberg B. The Interplay of Climate Change and Air Pollution on Health. Curr Environ Health Rep. 2017;4(4):504–13. 29080073 10.1007/s40572-017-0168-6 PMC5676805

[12] Xu R, Yu P, Abramson MJ, Johnston FH, Samet JM, Bell ML, . Wildfires, Global Climate Change, and Human Health. N Engl J Med. 2020;383(22):2173–81. 33034960 10.1056/NEJMsr2028985

[13] Ellis EC, Gauthier N, Klein Goldewijk K, Bliege Bird R, Boivin N, Díaz S, . People have shaped most of terrestrial nature for at least 12,000 years. Proc Natl Acad Sci U S A. 2021;118(17). 10.1073/pnas.2023483118 PMC8092386 33875599

[14] Tome J, Richmond HL, Rahman M, Karmacharya D, Schwind JS. Climate change and health vulnerability in Nepal: A systematic review of the literature since 2010. Glob Public Health. 2022;17(7):1406–19. 34061709 10.1080/17441692.2021.1924824

[15] Romps DM, Seeley JT, Vollaro D, Molinari J. Climate change. Projected increase in lightning strikes in the United States due to global warming. Science. 2014;346(6211):851–4. 25395536 10.1126/science.1259100

[16] OECD. Taming Wildfires in the Context of Climate Change2023.

[17] Ambrosino N, Fracchia C. Strategies to relieve dyspnoea in patients with advanced chronic respiratory diseases. A narrative review. Pulmonology. 2019;25(5):289–98. 31129045 10.1016/j.pulmoe.2019.04.002

[18] Prevalence and attributable health burden of chronic respiratory diseases, 1990–2017: a systematic analysis for the Global Burden of Disease Study 2017. Lancet Respir Med. 2020;8(6):585–96. 32526187 10.1016/S2213-2600(20)30105-3 PMC7284317

[19] Meng M, Wei R, Wu Y, Zeng R, Luo D, Ma Y, . Long-term risks of respiratory diseases in patients infected with SARS-CoV-2: a longitudinal, population-based cohort study. EClinicalMedicine. 2024;69:102500. 38389713 10.1016/j.eclinm.2024.102500 PMC10882104

[20] Yang IA, Jenkins CR, Salvi SS. Chronic obstructive pulmonary disease in never-smokers: risk factors, pathogenesis, and implications for prevention and treatment. Lancet Respir Med. 2022;10(5):497–511. 35427530 10.1016/S2213-2600(21)00506-3

[21] Yan ML, Li TT. A Review of the Interactive Effects of Climate and Air Pollution on Human Health in China. Curr Environ Health Rep. 2024;11(2):102–8. 38351403 10.1007/s40572-024-00432-z

[22] Afifa, Arshad K, Hussain N, Ashraf MH, Saleem MZ. Air pollution and climate change as grand challenges to sustainability. Sci Total Environ. 2024;928:172370. 38604367 10.1016/j.scitotenv.2024.172370

[23] Shindell D, Smith CJ. Climate and air-quality benefits of a realistic phase-out of fossil fuels. Nature. 2019;573(7774):408–11. 31534245 10.1038/s41586-019-1554-z

[24] Perera F, Ashrafi A, Kinney P, Mills D. Towards a fuller assessment of benefits to children’s health of reducing air pollution and mitigating climate change due to fossil fuel combustion. Environ Res. 2019;172:55–72. 30771627 10.1016/j.envres.2018.12.016

[25] Liang Y, Wu C, Wu D, Liu B, Li YJ, Sun J, . Vertical distributions of atmospheric black carbon in dry and wet seasons observed at a 356-m meteorological tower in Shenzhen, South China. Sci Total Environ. 2022;853:158657. 36096219 10.1016/j.scitotenv.2022.158657

[26] Fierce L, Bond TC, Bauer SE, Mena F, Riemer N. Black carbon absorption at the global scale is affected by particle-scale diversity in composition. Nat Commun. 2016;7:12361. 27580627 10.1038/ncomms12361 PMC5025768

[27] Zhang JJ, Samet JM. Chinese haze versus Western smog: lessons learned. J Thorac Dis. 2015;7(1):3–13. 25694813 10.3978/j.issn.2072-1439.2014.12.06 PMC4311077

[28] Li T, Chen C, Zhang M, Zhao L, Liu Y, Guo Y, . Accountability analysis of health benefits related to National Action Plan on Air Pollution Prevention and Control in China. PNAS Nexus. 2024;3(4):pgae142. 38689709 10.1093/pnasnexus/pgae142 PMC11060103

[29] Fisher S, Bellinger DC, Cropper ML, Kumar P, Binagwaho A, Koudenoukpo JB, . Air pollution and development in Africa: impacts on health, the economy, and human capital. Lancet Planet Health. 2021;5(10):e681–8. 34627472 10.1016/S2542-5196(21)00201-1

[30] Bauer SE, Im U, Mezuman K, Gao CY. Desert Dust, Industrialization, and Agricultural Fires: Health Impacts of Outdoor Air Pollution in Africa. J Geophys Res Atmos. 2019;124(7):4104–20.

[31] Ravishankara AR, David LM, Pierce JR, Venkataraman C. Outdoor air pollution in India is not only an urban problem. Proc Natl Acad Sci U S A. 2020;117(46):28640–4. 33139542 10.1073/pnas.2007236117 PMC7682420

[32] Anjum MS, Ali SM, Imad-Ud-Din M, Subhani MA, Anwar MN, Nizami AS, . An Emerged Challenge of Air Pollution and Ever-Increasing Particulate Matter in Pakistan; A Critical Review. J Hazard Mater. 2021;402:123943. 33254830 10.1016/j.jhazmat.2020.123943

[33] Yusuf S, Joseph P, Rangarajan S, Islam S, Mente A, Hystad P, . Modifiable risk factors, cardiovascular disease, and mortality in 155 722 individuals from 21 high-income, middle-income, and low-income countries (PURE): a prospective cohort study. Lancet. 2020;395(10226):795–808. 31492503 10.1016/S0140-6736(19)32008-2 PMC8006904

[34] Hegglin MI, Shepherd TG. Large climate-induced changes in ultraviolet index and stratosphere-to-troposphere ozone flux. Nat Geosci. 2009;2(10):687–91.

[35] Dai A. Increasing drought under global warming in observations and models. Nat Clim Chang. 2013;3(1):52–8.

[36] Hu YT, Wu QG, Hu AX, Schroeder S. Quantifying contributions of ozone changes to global and arctic warming during the second half of the twentieth century. Clim Dyn. 2023;61(3–4):1209–28.

[37] Fiore AM, Naik V, Leibensperger EM. Air quality and climate connections. J Air Waste Manag Assoc. 2015;65(6):645–85. 25976481 10.1080/10962247.2015.1040526

[38] Ball WT, Haigh JD, Rozanov EV, Kuchar A, Sukhodolov T, Tummon F, . High solar cycle spectral variations inconsistent with stratospheric ozone observations. Nat Geosci. 2016;9(3):206-U129.

[39] Dameris M. Climate change and atmospheric chemistry: how will the stratospheric ozone layer develop? Angew Chem Int Ed Engl. 2010;49(44):8092–102. 20922727 10.1002/anie.201001643

[40] Klobas JE, Hansen J, Weisenstein DK, Kennedy RP, Wilmouth DM. Sensitivity of Iodine-Mediated Stratospheric Ozone Loss Chemistry to Future Chemistry-Climate Scenarios. Front Earth Sci. 2021;9.

[41] Robinson SA, Revell LE, Mackenzie R, Ossola R. Extended ozone depletion and reduced snow and ice cover-Consequences for Antarctic biota. Glob Chang Biol. 2024;30(4):e17283. 38663017 10.1111/gcb.17283

[42] Cotrufo MF, Ranalli MG, Haddix ML, Six J, Lugato E. Soil carbon storage informed by particulate and mineral-associated organic matter. Nat Geosci. 2019;12(12):989.

[43] Yang L, Zhang H, Zhang X, Xing W, Wang Y, Bai P, . Exposure to Atmospheric Particulate Matter-Bound Polycyclic Aromatic Hydrocarbons and Their Health Effects: A Review. Int J Environ Res Public Health. 2021;18(4). 10.3390/ijerph18042177 PMC7926315 33672189

[44] Abdillah SFI, Wang YF. Ambient ultrafine particle (PM(0.1)): Sources, characteristics, measurements and exposure implications on human health. Environ Res. 2023;218:115061. 36525995 10.1016/j.envres.2022.115061

[45] Yang ZF, Demoz B, Delgado R, Sullivan J, Tangborn A, Lee PS. Influence of the transported Canadian wildfire smoke on the ozone and particle pollution over the Mid-Atlantic United States. Atmos Environ. 2022;273.

[46] Ebi KL, McGregor G. Climate change, tropospheric ozone and particulate matter, and health impacts. Environ Health Perspect. 2008;116(11):1449–55. 19057695 10.1289/ehp.11463 PMC2592262

[47] D’Amato G, Chong-Neto HJ, Monge Ortega OP, Vitale C, Ansotegui I, Rosario N, . The effects of climate change on respiratory allergy and asthma induced by pollen and mold allergens. Allergy. 2020;75(9):2219–28. 32589303 10.1111/all.14476

[48] Auffret AG, Svenning JC. Climate warming has compounded plant responses to habitat conversion in northern Europe. Nat Commun. 2022;13(1). 10.1038/s41467-022-35516-7 PMC9763501 36535960

[49] De Sario M, Katsouyanni K, Michelozzi P. Climate change, extreme weather events, air pollution and respiratory health in Europe. Eur Respir J. 2013;42(3):826–43. 23314896 10.1183/09031936.00074712

[50] Xu Q, Zhou Q, Chen J, Li T, Ma J, Du R, . The incidence of asthma attributable to temperature variability: An ecological study based on 1990–2019 GBD data. Sci Total Environ. 2023;904:166726. 37659541 10.1016/j.scitotenv.2023.166726

[51] Pacheco SE, Guidos-Fogelbach G, Annesi-Maesano I, Pawankar R, G DA, Latour-Staffeld P, . Climate change and global issues in allergy and immunology. J Allergy Clin Immunol. 2021;148(6):1366–77. 34688774 10.1016/j.jaci.2021.10.011

[52] Samarkandi OA, Khan AA, Alazmy W, Alobaid AM, Bashatah AS. The pulmonary consequences of sandstorms in Saudi Arabia: A comprehensive review and update. Am J Disaster Med. 2017;12(3):179–88. 29270961 10.5055/ajdm.2017.0272

[53] Park J, Kim HJ, Lee CH, Lee CH, Lee HW. Impact of long-term exposure to ambient air pollution on the incidence of chronic obstructive pulmonary disease: A systematic review and meta-analysis. Environ Res. 2021;194:110703. 33417909 10.1016/j.envres.2020.110703

[54] Pérez Velasco R, Jarosińska D. Update of the WHO global air quality guidelines: Systematic reviews - An introduction. Environ Int. 2022;170:107556. 36395555 10.1016/j.envint.2022.107556 PMC9720155

[55] Gao H, Wang K, Au WW, Zhao WS, Xia ZL. A Systematic Review and Meta-Analysis of Short-Term Ambient Ozone Exposure and COPD Hospitalizations. Int J Environ Res Public Health. 2020;17(6). 10.3390/ijerph17062130 PMC7143242 32210080

[56] McConnell R, Berhane K, Gilliland F, London SJ, Islam T, Gauderman WJ, . Asthma in exercising children exposed to ozone: a cohort study. Lancet. 2002;359(9304):386–91. 11844508 10.1016/S0140-6736(02)07597-9

[57] Fang XY, Huang SJ, Zhu YX, Lei J, Xu YY, Niu Y, . Short-term exposure to ozone and asthma exacerbation in adults: A longitudinal study in China. Front Public Health. 2023;10. 10.3389/fpubh.2022.1070231 PMC9854395 36684992

[58] Alman BL, Pfister G, Hao H, Stowell J, Hu XF, Liu Y, . The association of wildfire smoke with respiratory and cardiovascular emergency department visits in Colorado in 2012: a case crossover study. Environ Health. 2016;15. 10.1186/s12940-016-0146-8 PMC4893210 27259511

[59] Hamra GB, Guha N, Cohen A, Laden F, Raaschou-Nielsen O, Samet JM, . Outdoor Particulate Matter Exposure and Lung Cancer: A Systematic Review and Meta-Analysis. Environ Health Perspect. 2014;122(9):906–11. 24911630 10.1289/ehp/1408092 PMC4154221

[60] Makrufardi F, Manullang A, Rusmawatiningtyas D, Chung KF, Lin SC, Chuang HC. Extreme weather and asthma: a systematic review and meta-analysis. Eur Respir Rev. 2023;32(168). 10.1183/16000617.0019-2023 PMC10245140 37286218

[61] Dennekamp M, Abramson MJ. The effects of bushfire smoke on respiratory health. Respirology. 2011;16(2):198–209. 20920143 10.1111/j.1440-1843.2010.01868.x

[62] Shaposhnikov D, Revich B, Bellander T, Bedada GB, Bottai M, Kharkova T, . Mortality related to air pollution with the moscow heat wave and wildfire of 2010. Epidemiology. 2014;25(3):359–64. 24598414 10.1097/EDE.0000000000000090 PMC3984022

[63] Gupta P, Singh S, Kumar S, Choudhary M, Singh V. Effect of dust aerosol in patients with asthma. J Asthma. 2012;49(2):134–8. 22211448 10.3109/02770903.2011.645180

[64] O’Gorman PA. Sensitivity of tropical precipitation extremes to climate change. Nat Geosci. 2012;5(10):697–700.

[65] Hayes D Jr, Jhaveri MA, Mannino DM, Strawbridge H, Temprano J. The effect of mold sensitization and humidity upon allergic asthma. Clin Respir J. 2013;7(2):135–44. 22524711 10.1111/j.1752-699X.2012.00294.x

[66] Costabel U, Miyazaki Y, Pardo A, Koschel D, Bonella F, Spagnolo P, . Hypersensitivity pneumonitis. Nat Rev Dis Primers. 2020;6(1):65. 32764620 10.1038/s41572-020-0191-z

[67] Zou E, Worsham C, Miller NH, Molitor D, Reif J, Jena AB. Emergency Visits for Thunderstorm-Related Respiratory Illnesses Among Older Adults. JAMA Intern Med. 2020;180(9):1248–50. 10.1001/jamainternmed.2020.1672 PMC7418045 32777000

[68] Harun NS, Lachapelle P, Douglass J. Thunderstorm-triggered asthma: what we know so far. J Asthma Allergy. 2019;12:101–8. 31190900 10.2147/JAA.S175155 PMC6512777

[69] Davies JM, Thien F, Hew M. Thunderstorm asthma: controlling (deadly) grass pollen allergy. BMJ. 2018;360:k432. 29437642 10.1136/bmj.k432

[70] Rice MB, Li W, Wilker EH, Gold DR, Schwartz J, Zanobetti A, . Association of outdoor temperature with lung function in a temperate climate. Eur Respir J. 2019;53(1). 10.1183/13993003.00612-2018 PMC6903481 30578386

[71] Davis RE, Markle ES, Windoloski S, Houck ME, Enfield KB, Kang H, . A comparison of the effect of weather and climate on emergency department visitation in Roanoke and Charlottesville, Virginia. Environ Res. 2020;191:110065. 32827524 10.1016/j.envres.2020.110065 PMC7658034

[72] Tran HM, Chuang TW, Chuang HC, Tsai FJ. Climate change and mortality rates of COPD and asthma: A global analysis from 2000 to 2018. Environ Res. 2023;233:116448. 37352955 10.1016/j.envres.2023.116448

[73] Gonzalez-Garcia M, Barrero M, Maldonado D. Exercise Capacity, Ventilatory Response, and Gas Exchange in COPD Patients With Mild to Severe Obstruction Residing at High Altitude. Front Physiol. 2021;12:668144. 34220533 10.3389/fphys.2021.668144 PMC8249805

[74] Segers FJ, van Laarhoven KA, Huinink HP, Adan OC, Wösten HA, Dijksterhuis J. The Indoor Fungus Cladosporium halotolerans Survives Humidity Dynamics Markedly Better than Aspergillus niger and Penicillium rubens despite Less Growth at Lowered Steady-State Water Activity. Appl Environ Microbiol. 2016;82(17):5089–98. 27316968 10.1128/AEM.00510-16 PMC4988216

[75] Prester L. Indoor exposure to mould allergens. Arh Hig Rada Toksikol. 2011;62(4):371–80. 22202471 10.2478/10004-1254-62-2011-2126

[76] Agarwal R, Muthu V, Sehgal IS, Dhooria S, Prasad KT, Aggarwal AN. Allergic Bronchopulmonary Aspergillosis. Clin Chest Med. 2022;43(1):99–125. 35236565 10.1016/j.ccm.2021.12.002

[77] Agarwal R, Muthu V, Sehgal IS. Relationship between Aspergillus and asthma. Allergol Int. 2023;72(4):507–20. 37633774 10.1016/j.alit.2023.08.004

[78] Katotomichelakis M, Nikolaidis C, Makris M, Proimos E, Aggelides X, Constantinidis TC, . Alternaria and Cladosporium calendar of Western Thrace: Relationship with allergic rhinitis symptoms. Laryngoscope. 2016;126(2):E51–6. 26371953 10.1002/lary.25594

[79] Villanueva DM, Venkatesan B, Figueroa N. Cladosporium sphaerospermum as a Rare Cause of Pneumonia. Cureus. 2022;14(6):e26256. 35898384 10.7759/cureus.26256 PMC9308497

[80] Sharpe RA, Bearman N, Thornton CR, Husk K, Osborne NJ. Indoor fungal diversity and asthma: a meta-analysis and systematic review of risk factors. J Allergy Clin Immunol. 2015;135(1):110–22. 25159468 10.1016/j.jaci.2014.07.002

[81] Wang J, Li Y, Zhao P, Tian Y, Liu X, He H, . Exposure to Air Pollution Exacerbates Inflammation in Rats with Preexisting COPD. Mediators Inflamm. 2020;2020:4260204. 32454790 10.1155/2020/4260204 PMC7231193

[82] Zhao C, Wang Y, Su Z, Pu W, Niu M, Song S, . Respiratory exposure to PM2.5 soluble extract disrupts mucosal barrier function and promotes the development of experimental asthma. Sci Total Environ. 2020;730:139145. 32402975 10.1016/j.scitotenv.2020.139145

[83] Wang L, Cui Y, Liu H, Wu J, Li J, Liu X. PM2.5 aggravates airway inflammation in asthmatic mice: activating NF-κB via MyD88 signaling pathway. Int J Environ Health Res. 2023;33(6):563–74. 35227140 10.1080/09603123.2022.2041561

[84] McMichael AJ, Lindgren E. Climate change: present and future risks to health, and necessary responses. J Intern Med. 2011;270(5):401–13. 21682780 10.1111/j.1365-2796.2011.02415.x

[85] Balmes JR, Aris RM, Chen LL, Scannell C, Tager IB, Finkbeiner W, . Effects of ozone on normal and potentially sensitive human subjects. Part I: Airway inflammation and responsiveness to ozone in normal and asthmatic subjects. Res Rep Health Eff Inst. 1997;(78):1–37; discussion 81–99. 9387195

[86] Alexis NE, Lay JC, Hazucha M, Harris B, Hernandez ML, Bromberg PA, . Low-level ozone exposure induces airways inflammation and modifies cell surface phenotypes in healthy humans. Inhal Toxicol. 2010;22(7):593–600. 20384440 10.3109/08958371003596587 PMC3162473

[87] Stenfors N, Bosson J, Helleday R, Behndig AF, Pourazar J, Törnqvist H, . Ozone exposure enhances mast-cell inflammation in asthmatic airways despite inhaled corticosteroid therapy. Inhal Toxicol. 2010;22(2):133–9. 20044881 10.3109/08958370903005736

[88] Yan LI, Gong C, Ying L, Fu W, Liu S, Dai J, . PM2.5 affects establishment of immune tolerance in newborn mice by reducing PD-L1 expression. J Biosci. 2019;44(2). 31180054

[89] Liu H, Fan X, Wang N, Zhang Y, Yu J. Exacerbating effects of PM2.5 in OVA-sensitized and challenged mice and the expression of TRPA1 and TRPV1 proteins in lungs. J Asthma. 2017;54(8):807–17. 28102732 10.1080/02770903.2016.1266495

[90] Li R, Kou X, Tian J, Meng Z, Cai Z, Cheng F, . Effect of sulfur dioxide on inflammatory and immune regulation in asthmatic rats. Chemosphere. 2014;112:296–304. 25048919 10.1016/j.chemosphere.2014.04.065

[91] Wiegman CH, Li F, Ryffel B, Togbe D, Chung KF. Oxidative Stress in Ozone-Induced Chronic Lung Inflammation and Emphysema: A Facet of Chronic Obstructive Pulmonary Disease. Front Immunol. 2020;11:1957. 32983127 10.3389/fimmu.2020.01957 PMC7492639

[92] Ding H, Jiang M, Li D, Zhao Y, Yu D, Zhang R, . Effects of Real-Ambient PM(2.5) Exposure on Lung Damage Modulated by Nrf2(−/). Front Pharmacol. 2021;12:662664. 33967806 10.3389/fphar.2021.662664 PMC8104929

[93] Xia R, Fang N, Yang Y, Xu F, Zhang L, Ji S. PM2.5 promotes apoptosis of alveolar epithelial cells via targeting ROS/p38 signaling pathway and thus leads to emphysema in mice. Minerva Med. 2023;114(5):652–7. 32491296 10.23736/S0026-4806.20.06652-5

[94] Li N, Xiong R, Li G, Wang B, Geng Q. PM2.5 contributed to pulmonary epithelial senescence and ferroptosis by regulating USP3-SIRT3-P53 axis. Free Radic Biol Med. 2023;205:291–304. 37348684 10.1016/j.freeradbiomed.2023.06.017

[95] Havet A, Li Z, Zerimech F, Sanchez M, Siroux V, Le Moual N, . Does the oxidative stress play a role in the associations between outdoor air pollution and persistent asthma in adults? Findings from the EGEA study. Environ Health. 2019;18(1):90. 31665023 10.1186/s12940-019-0532-0 PMC6819357

[96] Ji JS, Xi D, Huang C. Building resilience in heatwaves. Nat Med. 2023;29(7):1613–4. 37464046 10.1038/s41591-023-02409-1

[97] Carlsten C, Salvi S, Wong GWK, Chung KF. Personal strategies to minimise effects of air pollution on respiratory health: advice for providers, patients and the public. Eur Respir J. 2020;55(6). 10.1183/13993003.02056-2019 PMC7270362 32241830

[98] Zhang W, Wang J, Chen B, Ji X, Zhao C, Chen M, . Association of multiple air pollutants with oxygen saturation during sleep in COPD patients: Effect modification by smoking status and airway inflammatory phenotypes. J Hazard Mater. 2023;454:131550. 37148791 10.1016/j.jhazmat.2023.131550

[99] Tikkakoski AP, Tikkakoski A, Sipilä K, Kivistö JE, Huhtala H, Kähönen M, . Exercise-induced bronchoconstriction is associated with air humidity and particulate matter concentration in preschool children. Pediatr Pulmonol. 2023;58(4):996–1003. 36530015 10.1002/ppul.26284

[100] Chen L, Cai M, Li H, Wang X, Tian F, Wu Y, . Risk/benefit tradeoff of habitual physical activity and air pollution on chronic pulmonary obstructive disease: findings from a large prospective cohort study. BMC Med. 2022;20(1):70. 35220974 10.1186/s12916-022-02274-8 PMC8883705

[101] Levy BS, Patz JA. Climate Change, Human Rights, and Social Justice. Ann Glob Health. 2015;81(3):310–22. 26615065 10.1016/j.aogh.2015.08.008

[102] Ravindra K, Goyal A, Mor S. Pollen allergy: Developing multi-sectorial strategies for its prevention and control in lower and middle-income countries. Int J Hyg Environ Health. 2022;242:113951. 35334435 10.1016/j.ijheh.2022.113951

[103] Soto-Martínez ME, Soto-Quiros ME, Custovic A. Childhood Asthma: Low and Middle-Income Countries Perspective. Acta Med Acad. 2020;49(2):181–90. 33189123 10.5644/ama2006-124.296

[104] Du M, Hu H, Zhang L, Liu W, Chu T, Wu G, . China county based COPD screening and cost-effectiveness analysis. Ann Palliat Med. 2021;10(4):4652–60. 33966413 10.21037/apm-21-812

[105] Bikomeye JC, Namin S, Anyanwu C, Rublee CS, Ferschinger J, Leinbach K, . Resilience and Equity in a Time of Crises: Investing in Public Urban Greenspace Is Now More Essential Than Ever in the US and Beyond. Int J Environ Res Public Health. 2021;18(16). 10.3390/ijerph18168420 PMC8392137 34444169

[106] Paavola J. Health impacts of climate change and health and social inequalities in the UK. Environ Health. 2017;16(Suppl 1):113. 29219089 10.1186/s12940-017-0328-z PMC5773866

[107] Arpin E, Gauffin K, Kerr M, Hjern A, Mashford-Pringle A, Barros A, . Climate Change and Child Health Inequality: A Review of Reviews. Int J Environ Res Public Health. 2021;18(20). 10.3390/ijerph182010896 PMC8535343 34682662

[108] Kim KH, Kabir E, Jahan SA. A Review of the Consequences of Global Climate Change on Human Health. J Environ Sci Health Part C Environ Carcinog Ecotoxicol Rev. 2014;32(3):299–318. 10.1080/10590501.2014.941279 25226222

[109] Tran HM, Tsai FJ, Lee YL, Chang JH, Chang LT, Chang TY, . The impact of air pollution on respiratory diseases in an era of climate change: A review of the current evidence. Sci Total Environ. 2023;898. 10.1016/j.scitotenv.2023.166340 37591374

[110] Kline O, Prunicki M. Climate change impacts on children’s respiratory health. Curr Opin Pediatr. 2023;35(3):350–5. 37057656 10.1097/MOP.0000000000001253

[111] Alahmad B, Khraishah H, Royé D, Vicedo-Cabrera AM, Guo Y, Papatheodorou SI, . Associations Between Extreme Temperatures and Cardiovascular Cause-Specific Mortality: Results From 27 Countries. Circulation. 2023;147(1):35–46. 36503273 10.1161/CIRCULATIONAHA.122.061832 PMC9794133

[112] Squires E. Effects of climate change on patients with respiratory and cardiovascular conditions. Nurs Stand. 2023;38(7):57–61. 10.7748/ns.2023.e12087 37259785

[113] O’Lenick CR, Winquist A, Chang HH, Kramer MR, Mulholland JA, Grundstein A, . Evaluation of individual and area-level factors as modifiers of the association between warm-season temperature and pediatric asthma morbidity in Atlanta, GA. Environ Res. 2017;156:132–44. 28342349 10.1016/j.envres.2017.03.021 PMC5633283

[114] Sheffield PE, Landrigan PJ. Global climate change and children’s health: threats and strategies for prevention. Environ Health Perspect. 2011;119(3):291–8. 20947468 10.1289/ehp.1002233 PMC3059989

[115] Nilsson M, Sie A, Muindi K, Bunker A, Ingole V, Ebi KL. Weather, climate, and climate change research to protect human health in sub-Saharan Africa and South Asia. Glob Health Action. 2021;14(sup1):1984014. 35377292 10.1080/16549716.2021.1984014 PMC8986241

[116] Wright CY, Garland RM, Norval M, Vogel C. Human health impacts in a changing South African climate. S Afr Med J. 2014;104(8):579–82. 26307804 10.7196/samj.8603

[117] Romanello M, Di Napoli C, Drummond P, Green C, Kennard H, Lampard P, . The 2022 report of the Lancet Countdown on health and climate change: health at the mercy of fossil fuels. Lancet. 2022;400(10363):1619–54. 36306815 10.1016/S0140-6736(22)01540-9 PMC7616806

[118] Chersich MF, Wright CY, Venter F, Rees H, Scorgie F, Erasmus B. Impacts of Climate Change on Health and Wellbeing in South Africa. Int J Environ Res Public Health. 2018;15(9). 10.3390/ijerph15091884 PMC6164733 30200277

